# Joint Impact of Clinical and Behavioral Variables on the Risk of Unplanned Readmission and Death after a Heart Failure Hospitalization

**DOI:** 10.1371/journal.pone.0129553

**Published:** 2015-06-04

**Authors:** Badri Padhukasahasram, Chandan K. Reddy, Yan Li, David E. Lanfear

**Affiliations:** 1 Center for health policy and health services research, Henry Ford Health System, Detroit, Michigan, United States of America; 2 Department of Computer Science, Wayne State University, Detroit, Michigan, United States of America; Fondazione G. Monasterio, ITALY

## Abstract

Most current methods for modeling rehospitalization events in heart failure patients make use of only clinical and medications data that is available in the electronic health records. However, information about patient-reported functional limitations, behavioral variables and socio-economic background of patients may also play an important role in predicting the risk of readmission in heart failure patients. We developed methods for predicting the risk of rehospitalization in heart failure patients using models that integrate clinical characteristics with patient-reported functional limitations, behavioral and socio-economic characteristics. Our goal was to estimate the predictive accuracy of the joint model and compare it with models that make use of clinical data alone or behavioral and socio-economic characteristics alone, using real patient data. We collected data about the occurrence of hospital readmissions from a cohort of 789 heart failure patients for whom a range of clinical and behavioral characteristics data is also available. We applied the Cox model, four different variants of the Cox proportional hazards framework as well as an alternative non-parametric approach and determined the predictive accuracy for different categories of variables. The concordance index obtained from the joint prediction model including all types of variables was significantly higher than the accuracy obtained from using only clinical factors or using only behavioral, socioeconomic background and functional limitations in patients as predictors. Collecting information on behavior, patient-reported estimates of physical limitations and frailty and socio-economic data has significant value in the predicting the risk of readmissions with regards to heart failure events and can lead to substantially more accurate events prediction models.

## Introduction

Rehospitalizations account for more than 30% of the 2 trillion annual cost of healthcare in the United States. Experts estimate that as many as 20% of all hospital admissions occur within 30 days of a previous discharge. Such rehospitalizations are not only expensive but are also potentially harmful, and most importantly, they are often preventable. Providing special care for a targeted group of patients who are at a high risk of rehospitalization can significantly improve the chances of avoiding rehospitalizations. However, such techniques have not been successful in practice due to a lack of understanding of the causes and risks of rehospitalization. Identifying patients at risk of rehospitalization can guide efficient resource utilization and is a cost-effective measure that can save millions of healthcare dollars each year. An important step towards preventing or better managing hospital readmissions is the identification of important prognostic factors to assess the risk of such events for individual patients through the construction of predictive models. This can enable us to identify important physiological targets or characteristic patient profiles that can allow for more focused medical or social interventions, reduce costs and improve the quality of healthcare provided by institutions. The objective of this work is to identify the patients with high risk of rehospitalization at the time of discharge using advanced regression methodology.

We collected data from a heart failure patient cohort for this study. Heart failure (HF) is a common and deadly disease [1] that affects over 5 million people within the US alone. Over 1 million patients are hospitalized with the primary diagnosis of heart failure annually and this condition contributes to over 200,000 deaths and expenditures exceeding 17 billion. HF is the most common cause of hospitalization in people over 65 and results in approximately 6.5 million hospital days annually. HF is also the largest contributor of unplanned readmissions and rehospitalizations and poses an enormous financial and social burden on the nation. Although some advances have been made in reducing mortality rates with respect to HF, rates of rehospitalization are on the rise and are estimated to be greater than 50% within six months of discharge. A significant portion of such readmissions are potentially preventable with timely, effective and adequate patient self-management. There have been many attempts to reduce avoidable readmissions in the HF population but none have yet proven broadly effective due to the difficulty in identifying the patients at highest risk in a timely way in order to focus interventions on this subgroup. One of the major problems in building robust and actionable models for predicting the risk of readmissions is the lack of complete information regarding what factors trigger the readmission. Electronic Health Records (EHR) presents a plethora of opportunities to decipher specific patient characteristics and make inferences about readmission for future patients. [2–3] However, this clinical data poses new challenges to the existing research and hence requires new models and methods to analyze and process it.

A large number of clinical variables have been established as important predictors of heart failure events. These include factors like blood pressure, smoking, medication intake, orthopnea,echocardiographic measures, cardiac biomarkers like natriuretic peptides, indicators of neurohormonal activation such as higher levels of circulating catecholamines and reninangiotensin system metabolites or lower levels of serum sodium as well as HF associated diagnoses like renal impairment, atrial fibrillation, ischemic heart disease, hypertension, diabetes and pulmonary diseases. Beyond these clinical factors, other factors related to patient behavior, socio-economic background and patient-reported estimates of functional limitations, disability and quality of life can also play a significant role in determining the probability of readmissions after heart failure.

Using Electronic Health Records (EHR) obtained from a large health system, namely the Henry Ford Health System (HFHS), we will first build regression models for readmission in patients hospitalized with a diagnosis of primary heart failure. Using a database of around 789 patients, we develop and study several regularized variants of the Cox proportional hazards regression models and random survival forests. Due to the difficulty in obtaining behavioral and socio-economic data, most of the hospitals and clinical studies do not consider such information. This is the reason why our study includes fewer patients though we have over 8,000 patients with only the clinical information. We demonstrate the predictive ability of the models using evaluation measures such as the c-index which is widely used in clinical applications. We also show that the variables selected by these regularized methods are clinically relevant based on the published medical literature about this problem. Finally, we show that adding behavioral data significantly improves the predictive performance according to the current clinical standards (c-index ~ 0.7) and is able to retrieve important biomarkers for predicting the future risk of rehospitalization.

### Objective

Providing special care for a targeted group of patients who are at a high risk of rehospitalization can significantly improve the chances of avoiding these events. However, such techniques have not been successful in practice due to a lack of understanding of the causes and risks of rehospitalization. Identifying patients at risk of rehospitalization can guide efficient resource utilization and is a cost-effective measure that can save millions of healthcare dollars each year. Despite the significance of this problem, not many researchers have thoroughly investigated it due to the inherent complexities involved in analyzing and estimating the predictive power of such complex data collected during the hospitalization of a patient. Effectively making predictions for this purpose will require a comprehensive set of predictors related to clinical covariates, medication use, behavior, socio-economic background and patient-reported estimates of quality of life. Using a variety of models under the Cox proportional hazards framework and through cross-validation we test the predictive value of clinical and medication use variables towards the risk of HF events. We perform similar analysis using a collection of variables related to patient behavior, their reported levels of disability, functional limitation/frailty and socio-economic status and check whether these kinds of variables can be significantly predictive of heart failure related readmissions. Lastly, we construct a joint model that makes use of information from all these different classes of variables and test its predictive value using real patient data.

## Materials and Methods

### Ethics approval

The Henry Ford Health System Institutional Review Board approved this study. Patient records and information was anonymized and de-identified prior to use in this analysis.

### Data Source

We will now describe all the data sources and factors that are being considered for our study. The data for this project will be comprehensively collected from the following sources of information that are collected at the Henry Ford Health System (HFHS) in south eastern Michigan. HFHS has the distinct advantage of serving a very diverse patient population, as well as advanced and readily available electronic data resources. Using administrative data resources, we identified all patients with a primary hospital discharge diagnosis of heart failure (9^th^ Edition/Revision International Classification of Diseases [ICD-9] codes used). Patients were selected based on the occurrence of clinical heart failure according to the Framingham criteria and who were members of the HAP (Health Alliance Plan) medical insurance with pharmaceutical benefits. Table 1 summarizes some sample characteristics of our study cohort. For our analysis, we chose a subset of 789 patients for which both clinical, medication use and behavioral variables data was available and for whom there was at least one readmission to the hospital after the initial visit date and the time (days) to the occurrence of such an event had been recorded. The entire set of variables that can potentially be important for readmission can be described under 2 broad groups. [4–5]

**Table 1:** Sample characteristics of the HFHS heart failure study cohort.

Characteristics	Patients with readmission events	Patients without readmission events
No. of samples	429	360
Average age	73.49 ± 12.63	71.35 ±11.03
No. of females (%)	176 (41.02%)	139 (38.61%)
Height	168.04 ± 17.62	168.78 ±13.29
Weight	92.34 ± 41.06	93.23 ± 51.93
Non-smokers (%)	173 (40.3%)	151 (41.9%)
Smokers (%)	256 (59.7%)	209 (58.1%)
Blood Pressure Systolic	131.65 + 27.64	131.98 + 23.17
Blood Pressure Diastolic	72.49 ± 12.76	72.96 ± 12.31
Heart failure type Class 0	286 (66.7%)	253 (70.3%)
Heart failure type Class 1	143 (33.3%)	107 (29.7%)
New York Heart Failure Association (NYHA) class: 0, 1, 2, 3, 4	53, 124, 138, 89, 25	6, 240, 76, 31, 7

#### 1. Clinical Variables, Medications and Procedures

The variables in this category include age, gender and ethnicity as well as other disease conditions associated with heart failure such as diabetes, hypertension, atrial fibrillation, myocardial infarction, and chronic lung disease.

According to a recent survey article [5], these conditions were included in a total of 24 out of 26 different readmission risk prediction models. Medication variables involve drugs such as Beta blockers, ACE (angiotensin-converting-enzyme) inhibitors and ARB (angiotensin receptor blockers). The procedures that are important include cardiac catheterization, hemodialysis and mechanical ventilation.

#### 2. Demographic, socio-economic, behavioral and quality of life variables

These variables include factors like education, household income, marital status, smoking status, alcohol consumption and patient reported estimates of frailty, general health and quality of life.

### Cox proportional hazards framework

In this section, we describe various survival models that can effectively handle both clinical and behavioral features to predict the risk of rehospitalization from a wide range of electronic medical records stored in multiple sources in a hospital setting. This will be one of the first studies to demonstrate the inherent predictive associations of clinical and behavioral variables for the heart failure readmissions problem. In our analysis, we will consider the Cox proportional hazards model and different variants of it to obtain the predictive power of the different groups of variables considered.

Cox proportional hazards is widely used in survival analysis. [6] Survival data consists of two important variables which are the observed time and censoring status. For the Cox regression, the notations are defined as follows. The *i* ^*th*^ sample will constitute the following triplet (*xi*, *yi*, *δi*) where *yi* is the observed time for *i* = 1, 2… *n* subjects. It is calculated as the minimum of the time to failure and censored times. *x*_*i*_ denotes the vector for feature representation for that sample. We will now provide the partial log likelihood for the Cox model.

$$l\left(\beta \right)\;=\;\frac{-2}{n}\left[\Sigma_{i=1}^{n}\delta_{i}x_{i}^{T}\beta \;-\;\delta_{i}log\left(\Sigma_{j\varepsilon R_{i}}\text{exp}\left(x_{i}^{T}\beta \right)\right)\;\right]$$

where *β* is a vector of regression coefficients. δ*i* is the censored status which is equal to 1 if *y*_*i*_ is the time to failure and δ_*i*_ = 0 if *y*_*i*_ is the censored time. *Ri* is the set of patient indices at risk for time *y*_*i*_. It consists of all those patients with index *j* for whom *y*_*j*_ ≥ *y*_*i*_. Because of its inherent nature of considering survival times and censoring, this Cox regression model has been used heavily by biostatistics researchers.

The primary reason for using regularized methods [7–10] is to effectively identify the most critical features that are contributing to the readmission risk and building a robust model that avoids the over-fitting problem. [11] To avoid the problem of over-fitting and avoiding the variables from taking extreme values, certain sparsity inducing norms are widely used to penalize the original partial log-likelihood function using L1 norm regularization term on the beta coefficients. There are three popular variations in the sparsity inducing norms, namely, lasso, ridge and elastic net. These variations add *Lp* norm penalty to the original objective function.

#### Cox Lasso

Lasso [12] is a *L*1 norm penalty which can select a few features while estimating the regression coefficient. In [13], the Lasso penalty was used along with the log-partial likelihood.

$${\hat{\beta }}_{lasso}\;=\;min\{-\frac{2}{N}[\sum_{j\;=\;1}^{N}\delta_{j}X_{j}\beta -\;\delta_{j}log(\sum_{i\epsilon R_{j}}e^{X_{i}\beta })]\;+\;\lambda \sum_{p\;=\;1}^{P}\left|\beta_{p}\right|\}$$

#### Ridge regression

This is a *L*2 norm regularization which tends to select all the correlated variables, and shrink their values towards each other. [14–16] The regression parameters of Cox-Ridge can be estimated by:

$${\hat{\beta }}_{ridge}\;=\;min\{-\frac{2}{N}[\sum_{j\;=\;1}^{N}\delta_{j}X_{j}\beta -\;\delta_{j}log(\sum_{i\epsilon R_{j}}e^{X_{i}\beta })]\;+\;\frac{\lambda }{2}\sum_{p\;=\;1}^{P}{\beta_{p}}^{2}\}$$

#### Elastic Net

The elastic net approach uses a convex combination of the L1 and squared L2 norm (ridge) penalty to obtain both sparsity and handle correlated feature spaces. [17] The logpartial likelihood function for the Cox-Elastic Net method [18] is given below:

$${\hat{\beta }}_{elastic\;net}=min\{-\frac{2}{N}[\overset{N}{\underset{j=1}{\sum }}\delta_{j}X_{j}\beta -\delta_{j}\text{log}(\underset{i\epsilon R_{j}}{\sum }e^{x_{i}\beta })]+\lambda [\alpha \overset{p}{\underset{p=1}{\sum }}\left|\beta_{p}\right|+\frac{1}{2}\left(1-\alpha \right)\overset{p}{\underset{p=1}{\sum }}\beta_{p}^{2}\}$$

where 0 ≤ α ≤ 1.

For all these regularized versions, the parameter λ ≥ 0 is used to adjust the influence of the penalty term. The optimal λ value is chosen via cross-validation.

#### Random Survival Forests

Random forest is an ensemble method designed specifically for tree structured prediction models. [19] In random survival forests, an extension of this methodology for right-censored survival data, the Nelson–Aalen estimator [20–21] is utilized to predict the cumulative hazard function (CHF). This estimator is defined as:

$$\hat{\Lambda }\left(t\right)\;=\;\sum_{t_{j}\leq t}\frac{d_{j}}{r_{j}}$$

where *d*_*j*_ is the number of deaths at time *t*_*j*,_ and *r*_*j*_ is the number of individuals at risk at *t*_*j*_. The main steps of this method are as follows: (1) Draw B bootstrap samples from the original dataset. (2) Grow a survival tree for each bootstrap sample, and ensure that in each terminal node the number of events occurred is no less than d (certain threshold value given by user). (3) Compute the CHF for each tree. For a test sample, the estimated ensemble CHF can then be calculated by taking the average of the corresponding CHF of the leaf node of each tree. [22]

#### CoxBoost

This was proposed in [23–24] to estimate parameter vector (*β*) in the Cox proportional hazards model. In each boosting step, the CoxBoost adaptively selects a flexible subset of covariates to update the corresponding parameters. In the *k* ^*th*^ boosting step, the Newton-Raphson step will be separately used for *g*_*k*_ predetermined candidate sets of covariates and the corresponding elements of *β* will be updated based on the candidate set which maximizes the improvement of the overall fit of the log-partial likelihood. Let us denote the chosen set using Φ, the updated estimated coefficient ${\hat{\beta }}^{\left(k\right)}$ of *k* ^*th*^ boosting step can be calculated as:

β^(k)=β^j(k-1)+Υ^j(k)ifj∈Φβ^j(k-1)ifj∈Φj=1,…,P

where ${\hat{\Upsilon }}_{j}^{\left(k\right)}$ is the element of the Newton-Raphson updating in *k* ^*th*^ boosting step. In addition, the chosen set Φ will not be considered as candidate set in the next boosting step. Thus, in the (*k* + 1)^*st*^ boosting step, *β* will be updated based on the remaining (*g*_*k*_ -1) predetermined candidates sets of covariates.

### Concordance Index

C-index, or the concordance probability [24–25], is one of the most commonly used evaluation method in survival analysis. Consider a pair of bivariate observations $\left(y_{1},\;{\hat{y}}_{1}\right)$ and $\left(y_{2},\;{\hat{y}}_{2}\right)$, where *y*_i_ is the actual observation, and ${\hat{y}}_{i}$ is the predicted value. The concordance probability is defined as:

$$c\;=\;Pr({\hat{y}}_{1}>{\hat{y}}_{2}|y_{1}>y_{2})$$

The Cox-based models and random survival forests predict the hazard ratio rather than the event time directly. Hence, a patient with a lower hazard ratio will survive longer. The c-index can be calculated by:

$$c\;=\;\frac{\sum_{i<j}I\left(y_{i}<y_{j}\right)I\left({\hat{\eta }}_{i}>{\hat{\eta }}_{j}\right)\delta_{i}+I\left(y_{j}<y_{i}\right)I\left({\hat{\eta }}_{j}>{\hat{\eta }}_{i}\right)\delta_{j}}{\sum_{i<j}I\left(y_{i}<y_{j}\right)\delta_{i}+I\left(y_{j}<y_{i}\right)\delta_{j}}$$

where *i*,*j = 1*,*2*,*…*,*n*,*I*() is the indicator function, and $\hat{\eta }$ is the predicted values. Here *n* is the number of samples considered for the study.

## Results

We used the Harrell’s concordance-index (c-index) [24] as our metric for clinical validation. The c-index is a measure of separation of 2 survival distributions that is widely used to measure prediction performance. We applied 4 different variants of the Cox model namely: Cox-Lasso, Cox-Ridge regression, Cox-Elastic net regression and Cox-Boost to predict HF events. In addition to the Cox model, we also used a non-parametric method of random survival forests to predict the occurrence of heart failure events. 10 fold cross-validation was used for all approaches to calculate concordance index. We applied these various approaches to 3 sets of variables available in our cohort: 1.) 123 Clinical and medication use variables 2.) 60 Behavioral, socio-economic and quality of life variables 3.) Groups 1 and 2 combined (183 variables).

Table 2 summarizes the results obtained for these analyses. We can clearly see that the joint model involving all 183 variables available in our cohort significantly outperforms models that include only a subset of these variables belonging to either Groups 1 or 2 as described previously. In most cases, we can see that Group 2 is doing slightly better than Group 1, but the combined set is providing much better results indicating that clinical/medication use and behavioral/quality of life variables contain complementary information about the patient’s condition.

**Table 2:** Model performance based on concordance index.

Model	Clinical	Behavioral	Clinical + Behavioral	P value
	p = 123	p = 60	p = 183	
Cox	0.6143	0.6376	0.6590	<0.00001
Cox Lasso	0.6246	0.6478	0.6768	<0.00001
Cox Ridge	0.6389	0.6377	0.6752	<0.00001
Cox Elastic Net	0.6318	0.6523	0.6813	<0.00001
CoxBoost	0.6319	0.6575	0.6901	<0.00001
Random Survival Forests	0.6660	0.6471	0.6922	<0.00001

### Top ranked factors for predicting the risk of reoccurrence of heart failure events

The joint model includes variables from 2 broad groups namely 1) Clinical, physiological and medication use variables and medical procedures and 2) Socioeconomic, demographic, behavioral and patient reported measures of disability, frailty and quality of life variables. From Table 2, we can clearly see that the joint model that includes both these classes of variables significantly outperforms model that only include a subset of categories. To identify the most important variables contributing to the joint predictive model, we determined the top 23 variables based on the absolute value of effect size estimates as determined by the CoxBoost method. The most important variables from Groups 1 and 2, their effect sizes and the fraction of patients experiencing readmissions for different values of the important variables are shown in Tables 3 and 4 respectively.

**Table 3:** Effect size estimates, fraction of patients with events (F) and number of Samples (N) for most important clinical variables (n = 789).

Variable	Class 1	Class 2	Beta	Class 1	Class 1	Class 2	Class 2
				F	N	F	N
Cardiomegaly	False	True	0.040	0.43	207	0.58	582
Site enrolled	Hospital	Clinic	0.384	0.50	729	0.99	60
Congestive Heart Failure	None	Exacerbation	0.089	0.51	749	1.00	40
Heart attack last 30 days	False	True	0.046	0.53	778	1.00	11
Implanted cardioverter defibrillator	False	True	0.051	0.50	648	0.60	141
Smokes	False	True	0.010	0.53	324	0.55	465
ACE inhibitor	False	True	0.214	0.52	760	1.00	29
Drug ARB	False	True	-0.044	0.54	787	1.00	2
Drug Aldo Ant	False	True	-0.053	0.54	784	1.00	5
Beta Blocker	False	True	0.094	0.51	743	1.00	46
Loop diuretic	False	True	0.013	0.53	756	1.00	33
Nitrates	False	True	-0.119	0.53	780	1.00	9
Hydralazine	False	True	-0.013	0.53	780	1.00	9

**Table 4:** Effect size estimates, fraction of patients with events (F) and Number of Samples (N) for most important behavioral variables (n = 789).

Variable	Categories	Beta	1	2	3	4	5	6	7
			F	F	F	F	F	F	F
			N	N	N	N	N	N	N
Limited by fatigue	1:All time, 2:Several a day, 3: > = once a day, 4:> = 3 times a week, 5:1–2 times a week, 6:<once a week, 7:never	-0.068	0.68	0.76	0.65	0.64	0.64	0.54	0.33
			70	93	93	76	94	87	276
Limitation in walking	1:Extreme, 2:Quite a bit, 3:Moderately, 4:Slightly, 5:Not at all, 6:Other/None	-0.059	0.82	0.80	0.74	0.54	0.38	0.74	
			50	85	92	160	375	27	
Changes in heart failure symptoms	1:Worse, 2: slightly worse, 3:no change, 4:slightly better, 5:Better, 6: None	-0.114	0.96	0.70	0.56	0.72	0.66	0.31	
			28	86	316	78	45	236	
Swelling causing botheration	1:Extreme, 2:Quite a bit, 3:Moderately, 4:Slightly, 5:Not at all,6:Other/None	-0.064	0.76	0.93	0.78	0.66	0.59	0.42	
			21	33	56	91	130	450	
Bother by fatigue	1:Extreme, 2:Quite a bit, 3:Moderately,4:Slightly, 5:Not at all, 6:Other/None	-0.044	0.80	0.78	0.60	0.59	0.56	0.31	
			60	84	112	203	82	248	
Sleep sitting	1:every night, 2:> = 3 a week, 3:1–2 times a week, 4:<once a week, 5:never	0.023	0.63	0.60	0.55	0.76	0.51		
			87	30	35	26	601		
General health	1:Excellent, 2:Very Good, 3: Good, 4:Fair, 5:Poor	0.125	0.21	0.35	0.48	0.66	0.76		
			42	129	256	266	96		
Limitation moderate activities	1: Limited a lot, 2: Limited a little, 3: Not limited at all	-0.011	0.71	0.55	0.36				
			223	337	229				
Alcohol per day	1:0 drinks, 2:1–2 drinks, 3: 3–4 drinks, 4:> = 5 drinks	-0.032	0.60	0.46	0.45	0.50			
			428	303	44	14			

## Discussion

We have utilized 6 different kinds of algorithms for predicting hospital readmissions related to heart failure events using a comprehensive set of variables including clinical, medication use,behavioral, socio-economic and measures of quality of life based on patient-reported measures of functional limitations and frailty. In particular, we used the standard Cox model as well as four different methods based on the Cox proportional hazards framework and regularization to predict the reoccurrence of heart failure events in our cohort. In addition, we have also utilized the nonparametric approach of random survival forests for comparison. All of the methods indicated that combining different categories of variables leads to more accurate prediction models than making use of clinical variables alone or behavioral and socio-economic variables alone. We observed a significant increase in c-index of around 0.03–0.04 when combining all the variables as compared to models that only use variables of a particular category.

We used three different sets of variables when constructing prediction models based on the six different methods mentioned above: i) Clinical and medication use variables ii) Behavioral, socio-economic factors and patient quality of life estimates iii) Variables from i) and ii) used jointly. For all three sets of variables we measured the c-index for 6 different algorithms. We found that in all scenarios the c-index obtained based on variable set iii) was substantially higher than the c-index obtained based on prediction models constructed from sets i) and ii). In summary, all of the methods used in this study indicated that predictive models that combine different categories of variables are more accurate than those that make use of clinical, physiological and medication use variables only or behavioral and socio-economic factors alone (increase in c-index of around 0.03–0.04). Formal statistical tests assuming normality indicated that these differences are highly statistically significant.

### Clinical impact of these findings

Despite dramatic medical and therapeutic advances to improve patient outcomes in the last 20 years, unplanned readmission rates continue to remain high for patients with heart failure.

Such events are complex and multi-factorial and can be influenced by a wide variety of factors including physiological, clinical and socio-economic factors, medication nonadherence, dietary indiscretions and lack of low sodium foods, drug and alcohol abuse and patient-reported levels of disability, wellness and quality of life. [26–27] Robust, actionable and data-based plans to reduce readmission rates are underdeveloped because not many trials have focused on post-discharge outcomes as well as due to disparate conclusions arising from different studies regarding the efficacy of disease management strategies. Therefore, it is important to construct models based on the best evidence in each health care system to reduce readmission rates of HF patients. [28]

The HF patient cohort at the Henry Ford Health System provides a valuable data source to assess the performance of different predictive models for HF-related readmissions and to better understand the important risk factors underlying these events. Models like the one presented in this study can be used to identify physiological targets (e.g. congestion, high blood-pressure, cardiac abnormalities such as coronary artery disease, atrial fibrillation and noncardiac comorbidities such as chronic obstructive pulmonary disease (COPD) and renal dysfunction) and characteristic profiles of patients at high risk of early readmissions, leading to targeted interventions and proactive care management programs. These can help improve their quality of care and functional status while reducing costs associated with HF-related rehospitalizations. [29–31] Interventions can take the form of comprehensive post-discharge planning, delayed discharge from hospital, early follow-up, greater follow-ups in the form of phone calls and home visits, telemonitoring and home weight monitoring [32–33], patient education and recommending caretakers and family members to become more watchful with regards to the health status of such patients. On the other hand, intensive monitoring steps may be avoided for patients with low risk for reoccurrence of heart failure events.

## Conclusions

Behavioral and socio-economic factors as well as knowledge of patient-reported quality of life and disability measures can substantially improve the accuracy of predicting unplanned readmissions in HF patients when used jointly with clinical and medication use variables available from electronic health records. The joint model that includes all such factors outperformed models that include only one a subset of these variables for both the Cox proportional hazards framework as well as for a non-parametric approach (random survival forests). Collecting information on behavior, patient-reported estimates of physical limitations and frailty and socio-economic data for HF patients has significant value in predicting the risk of HF-related readmissions and may lead to more effective and targeted interventions.
